# Homoharringtonine demonstrates a cytotoxic effect against triple-negative breast cancer cell lines and acts synergistically with paclitaxel

**DOI:** 10.1038/s41598-022-19621-7

**Published:** 2022-09-19

**Authors:** Riley Plett, Paul Mellor, Stephanie Kendall, S. Austin Hammond, Aren Boulet, Kristine Plaza, Frederick S. Vizeacoumar, Franco J. Vizeacoumar, Deborah H. Anderson

**Affiliations:** 1 Department of Anatomy, Physiology and Pharmacology, University of Saskatchewan, 107 Wiggins Road, Saskatoon, SK S7N 5E5 Canada; 2 Cancer Research, University of Saskatchewan, 107 Wiggins Road, Saskatoon, SK S7N 5E5 Canada; 3 Department of Biochemistry, Microbiology and Immunology, University of Saskatchewan, 107 Wiggins Road, Saskatoon, SK S7N 5E5 Canada; 4 Department of Pathology, Cancer Group, College of Medicine, University of Saskatchewan, Saskatoon, SK S7N 5E5 Canada; 5 Cancer Research, Saskatchewan Cancer Agency, 107 Wiggins Road, Saskatoon, SK S7N 5E5 Canada

**Keywords:** Breast cancer, Cancer therapy

## Abstract

The lack of targeted therapies for triple-negative breast cancer (TNBC) contributes to their high mortality rates and high risk of relapse compared to other subtypes of breast cancer. Most TNBCs (75%) have downregulated the expression of CREB3L1 (cAMP-responsive element binding protein 3 like 1), a transcription factor and metastasis suppressor that represses genes that promote cancer progression and metastasis. In this report, we screened an FDA-approved drug library and identified four drugs that were highly cytotoxic towards HCC1806 CREB3L1-deficient TNBC cells. These four drugs were: (1) palbociclib isethionate, a CDK4/6 inhibitor, (2) lanatocide C (also named isolanid), a Na+/K+-ATPase inhibitor, (3) cladribine, a nucleoside analog, and (4) homoharringtonine (also named omacetaxine mepesuccinate), a protein translation inhibitor. Homoharringtonine consistently showed the most cytotoxicity towards an additional six TNBC cell lines (BT549, HCC1395, HCC38, Hs578T, MDA-MB-157, MDA-MB-436), and several luminal A breast cancer cell lines (HCC1428, MCF7, T47D, ZR-75-1). All four drugs were then separately evaluated for possible synergy with the chemotherapy agents, doxorubicin (an anthracycline) and paclitaxel (a microtubule stabilizing agent). A strong synergy was observed using the combination of homoharringtonine and paclitaxel, with high cytotoxicity towards TNBC cells at lower concentrations than when each was used separately.

## Introduction

Breast cancer is the most common cancer in women, with a prevalence of 11.7% globally^1^, but rises to 28% in Canada^2^. Women with metastatic breast cancer have a disheartening 5-year survival rate of only 28%^3^. There are three main subtypes of breast cancer categorized by expression of their estrogen receptor (ER), progesterone receptor (PR) and human epidermal growth factor receptor (HER2)^4^. Luminal breast cancers (65–75%) express estrogen and/or progesterone receptors and are typically treated with anti-estrogens, such as aromatase inhibitors (e.g., anastrozole), selective estrogen receptor modulators (e.g., tamoxifen) and selective estrogen receptor degraders (e.g., fulvestrant)^5^. HER2+ breast cancers (10–15%) overexpress the HER2 receptor and are treated with antibodies to HER2 such as trastuzumab^5^. Triple-negative breast cancers (TNBCs; 15–20%) lack the three receptors that define the other subtypes. Since they are missing these molecular targets, TNBCs are treated with various combinations of general cytotoxic chemotherapy agents as the primary treatment^6^. Thus, there is an unmet clinical need to develop treatments for TNBC that are both effective and have fewer toxic side effects.

CREB3 (cAMP-responsive element binding protein 3)-family transcription factors have important roles in tissue development, lipid metabolism, protein secretion and tumorigenesis^7^. Their normal roles are to provide cell-type specific functions to different tissues to maintain cellular homeostasis in response to cell stress by regulating cell secretory capacity and cell specific cargos^8–10^. They are endoplasmic reticulum transmembrane proteins and in response to cell stress, they traffic to the Golgi complex to be cleaved by Golgi-resident S1P and S2P proteases to generate active cytosolic transcription factors that enter the nucleus to regulate gene expression^7^.

We have been studying a member of CREB3-family, CREB3L1 (cAMP-responsive element binding protein 3 like 1), a transcription factor responsible for repressing the expression of genes that promote breast cancer progression and metastasis^11,12^. CREB3L1 expression is frequently upregulated in early breast cancers, but its expression is significantly reduced in more advanced and metastatic breast cancers due to epigenetic silencing^12,13^. Loss of CREB3L1 expression is associated with poor prognosis and reduced patient survival times in luminal A (ER+, HER2−) and TNBCs^12,14^.

Overall, ~ 30% of breast cancers lack CREB3L1 expression^12,15^. CREB3L1-deficient breast cancers are typically the more advanced metastatic breast tumors and include ~ 75% of TNBCs^15^. We have shown that CREB3L1 loss directly contributes to the metastatic phenotype of breast cancer cells, using in vitro cell-based assays and animal models of breast cancer^11,12,15^. Re-expression of CREB3L1 in CREB3L1-deficient breast cancer cells significantly decreases metastatic properties (of growth in soft agar, migration, invasion)^11,15^. Consistent with these results, poorly metastatic CREB3L1-expressing cells can be converted to more metastatic phenotypes by CREB3L1 knockdown^11^.

We have also characterized tumor formation and metastasis using a syngeneic rat mammary tumor model^11^. CREB3L1-deficient cells formed tumors at high frequencies, and most had lymph node metastases. The same cells engineered to stably express CREB3L1 formed primary tumors at a reduced frequency on day 30 and by day 60 many of these regressed to a nearly undetectable size and none formed metastases^11^. Blood vessel formation within the regressing CREB3L1-expressing tumors was significantly decreased compared to tumors from CREB3L1-deficient animals (p < 0.001)^11^. These results strongly suggest that CREB3L1 blocks angiogenesis, which is necessary for large tumors to survive and plays a key role in metastasis suppression. Additionally, re-expression of CREB3L1 in a mouse xenograft model of human TNBC similarly showed reduced tumor progression and lung metastases, as compared to the CREB3L1-deficient parental TNBC, further supporting the role of CREB3L1 as a metastasis suppressor^15^.

In this project, we have used this newly identified molecular signature of CREB3L1-deficiency to identify the most effective new drug(s) for the treatment of CREB3L1-deficient metastatic TNBCs. Our approach was to identify FDA-approved drugs that are preferentially more cytotoxic towards CREB3L1-deficient TNBCs, as compared to the same cells re-expressing CREB3L1, and then to test the most effective drugs in combination with two frequently used chemotherapy agents, doxorubicin and paclitaxel.

## Methods

### Cell lines and cell culture

Cell lines used included TNBC cell lines (HCC1806, BT549, HCC1395, HCC38, Hs578T, MDA-MB-157, MDA-MB-436), luminal A cell lines (HCC1428, MCF7, T47D, ZR-75-1) and non-tumorigenic breast cell line (MCF10A), all obtained from the American Type Culture Collection (ATCC, Gaithersburg, MD). Cells were authenticated by the supplier (http://www.ATCC.org) and cultured as recommended by ATCC for less than six months from the time of resuscitation. HCC1806, HCC1428 and T47D cells stably expressing triple hemagglutinin (HA)-tagged CREB3L1 (HA-CREB3L1) have been described and characterized previously^15^.

To visualize and count cells for the primary high-throughput drug screen, HCC1806 cells and HCC1806 + HACREB3L1 cells were labeled by the stable expression of red-fluorescent protein (RFP). Each cell line was transduced with an RFP-encoding lentivirus (pLJM5-RFP-hygro)^16^ and selected for 2 weeks in hygromycin (12.5 µg/mL; 10687-010, Invitrogen, Waltham, MA). The RFP-labeled cells growing in hygromycin-containing media grew more slowly than their unlabeled counterparts, but had similar doubling times to each other, though their plating efficiencies were different. There was no difference in the proliferation rate between HCC1806 and HCC1806 + HACREB3L1 cells (both RFP-expressing and non-RFP-expressing), though they also had different plating efficiencies.

### Primary drug screen

A high-throughput drug screen of an FDA-approved drug library (1,818 compounds; TargetMol, Boston, MA, L1000) was carried out on HCC1806 ± HA-CREB3L1 cells at the Phenogenomic Imaging Centre of Saskatchewan. Briefly, in parallel, the RFP-labeled cell types were seeded into 384-well black-walled plates (142761, NUNC), HCC1806 cells (1300 cells/well) and HCC1806 + HA-CREB3L1 (2600 cells/well) in a total volume of 50 µL/well. Different starting cell numbers were used to take into account differences in their plating efficiencies noted above. Cells were allowed to attach and grow at 37 °C and 5% CO_2_. The following day drugs were added to each well (0.2 µL; 250 µM stock in dimethyl sulfoxide (DMSO)) giving a final concentration of 1 µM using a Biomek FX liquid handling system (A31843). Control DMSO wells were also included to control for impacts independent of the test drug. Live cells were imaged both prior to and after drug addition, and then each day for a total of 4 days using an automated imaging fluorescence microscope system, ImageXpress Micro XLS Widefield (Molecular Devices). The number of RFP-labeled cells remaining attached to the well was counted over time. After 4 days of drug treatment, cell viability (%) was determined for each drug test well relative to the corresponding DMSO control well.

### Secondary drug screen

Drugs selected from the primary screen (47) were independently sourced and reconstituted using the appropriate solvent (DMSO, dimethylformamide (DMF), ethanol (EtOH) or phosphate-buffered saline (PBS)) as indicated by the supplier (Cedarlane, Burlington, ON; Table S1). Drugs were generally reconstituted to 10 mM, except for a few that had a lower solubility, and all reconstituted drugs were stored at – 80 °C. To use, drugs were diluted into media (to 1 µM) to maintain a low solvent concentration (≤ 0.1%) and solvent control samples were generated and tested in parallel.

For secondary drug testing, unlabeled HCC1806 (500 cells/well) and HCC1806 + HA-CREB3L1 cells (600 cells/well) were seeded in triplicate wells in sterile 384-well black-walled optical bottom plate (6007558, PerkinElmer, Woodbridge, ON) using an ASSIST PLUS pipetting robot and a 16-channel VIAFLO pipette (4505 and 4642, INTEGRA Biosciences AG, Hudson, NH) in 50 µL. The doubling time of HCC1806 cells was not significantly impacted by HA-CREB3L1 expression^15^, though their initial plating efficiencies differed slightly. Cells were allowed to attach and grow at 37 °C and 5% CO_2_. The following day the media was carefully removed from each well and replaced with drug-containing media in triplicate wells, again using the ASSIST PLUS. After 4 days of drug treatment cells were stained with Hoechst and ImageIT Dead Green to quantify total and live cells as detailed below.

### Cell staining and viability determination

To quantify total and dead cells, they were stained with media containing Hoechst 33324 dye (5 μM; ThermoFisher Scientific, Saskatoon, SK, 62249) and ImageIT Dead Green dye (100 nM; ThermoFisher Scientific, Saskatoon, SK, I10291) for 30 min at 37 °C with 5% CO_2_. Images of each well of the 384-well plates were acquired using a Thermo Scientific™ CellInsight™ CX7 High Content Screening (HCS) Platform. Nine fields of view were captured using a 10× (0.4 NA) air objective lens. Images were analyzed using Thermo Scientific™ HCS Studio 3 Cell Analysis Software using the Spot Detector bio-application (Cellomics). Total cells were identified and counted by their Hoechst-stained nuclei at 386 nm (blue) channel 1 (Ch1) and dead cells were quantified from the ImageIT Dead Green on the 485 nm wavelength (green) channel 2 (Ch2). The total live cell count for each well containing a drug (determined from Ch1), was normalized to the total live cell count for each control well to account for any dead cells that had lifted off of the plate in test wells. The test well total live cell count was divided by the average of the corresponding solvent control total live cell count, giving the percentage cell viability (% viability). The mean ± standard error of the mean (SEM) of the % viability of each set of triplicate wells was determined.

### EC_50_ determinations—drug titration experiments

Drug titration experiments were carried out as described for the secondary drug screens, except a range of drug concentrations were tested. Typically, drugs were serially diluted in media 1:3 over the range of drug concentrations as noted in the text and figure legends (e.g., 0–333 nM, 0–2 μM, 0–9 μM, 0–50 μM), using the appropriate solvent control wells. Some drugs had to be further diluted to even lower concentrations, typically using 1:2 dilutions to best define the dose response curve. EC_50_ values were determined using PRISM software (GraphPad, San Diego, CA, v9.2.0) using a non-linear curve fit. A student's t-test was used to determine statistically significant differences between the EC_50_ values for the two cell lines. Significance was defined as p < 0.05. EC_50_ values of drugs that were more cytotoxic to the CREB3L1 deficient cells were determined from three independent experiments, each typically containing triplicate measurements. EC_50_ values of drugs that were equally or less cytotoxic to CREB3L1 deficient cells were determined from one experiment, typically containing triplicate measurements.

For drug titration experiments involving additional cell lines, plating efficiencies were determined for each cell line so that an appropriate number of cells were used to have significant numbers of cells to count in control (solvent only) wells over the 5 day experiment.

### Synergy experiments

Combination drug titration experiments were performed to determine potential selective cytotoxic sensitivity and synergy of the top 4 compounds with hallmark chemotherapeutic reagents, doxorubicin (A3966, ApexBio, made up in DMSO) and paclitaxel (10461, Cayman Chemical, Ann Arbor, MI, made up in DMSO) in the paired TNBC cell lines CREB3L1-deficient HCC1806 cells and CREB3L1 re-expressing HCC1806 + HACREB3L1 cells. A cost-effective and robust cross-design format was used which combines a background drug with a foreground drug^17^. The background drug is used at its EC_50_ value, whereas the foreground drug is tested across a range of doses. Each drug in the combination is tested as both a background drug and a foreground drug. Cells were plated, treated and cell viability was assessed as described previously. Results from 3 titrations were analyzed for each drug combination.

To determine the possible synergy of the combined drugs SynergyFinder software (version 2.0.11) was used with the default settings in R (version 3.6.1)^18^. The SynergyFinder package adjusted the % Viability (response) values input to % Cytotoxicity values. There were no other adjustments made to the data. Both monotherapy and combination data were input into the program. A delta score of 0 indicated no interaction or additivity (white in the synergy landscape). A negative delta score indicated antagonism (green in the synergy landscape). A positive delta score indicated synergy (red in the synergy landscape), with values > 10 indicating significant synergy.

### Statistical analyses

All results were expressed as the mean ± standard errors from at least 3 independent experiments, unless otherwise indicated. The significance of changes was assessed by the application of a student's t-test significance considered at p < 0.05 (Microsoft Excel v16.56).

## Results

### High-throughput drug screen and EC_50_ determinations

**Figure 1:**
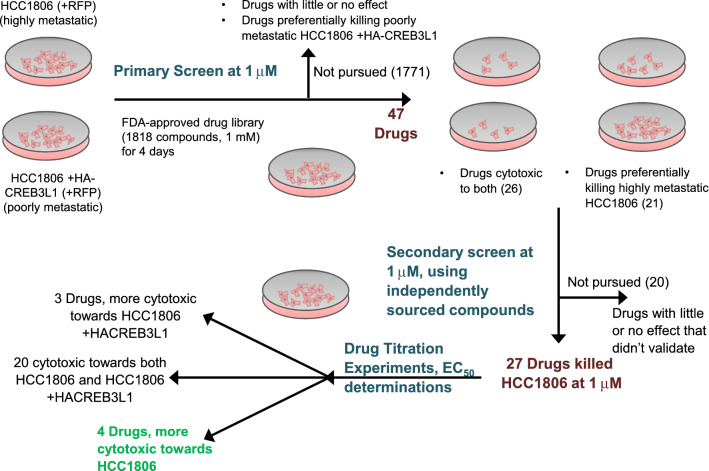
Primary and secondary drug screen schematic. Details are described within the text.

To identify drugs that are selectively cytotoxic towards more highly metastatic TNBC cells we used a matched pair of cell lines: highly metastatic CREB3L1-deficient HCC1806 cells and poorly metastatic HCC1806 + HA-CREB3L1 cells^15^. To help visualize and count cells, each cell line was stably transfected to express red fluorescent protein (RFP). A high-throughput drug screen was performed using an FDA-approved library of 1,818 compounds at an initial test concentration of 1 µM for 4 days of drug treatment (Fig. 1). Two groups of drugs were advanced to secondary screening: (1) drugs that showed 40% or more cytotoxicity towards the metastatic CREB3L1-deficient HCC1806 cells (21 drugs), and (2) drugs that killed both cell lines with similar efficacy at this single test concentration (1 µM) in case they might show selective killing of HCC1806 when tested at lower concentrations (26 drugs) (see Additional File 1: Table S1). A total of 47 drugs were identified as either more cytotoxic towards CREB3L1-deficient HCC1806 cells, as compared to HCC1806 + HA-CREB3L1 cells, or very cytotoxic towards both cell lines (see Additional File 1: Table S1).

The 47 compounds identified from the drug library were purchased from independent commercial sources and a validation experiment was carried out. All subsequent cytotoxicity assays used non-RFP-labeled cells and counted total cells (Hoechst 33324 dye), subtracting dead cells (ImageIT Dead Green dye), to determine cell viability after 4 days of drug treatment. The initial validation experiment tested the cytotoxicity of each of the 47 drugs towards HCC1806 ± HA-CREB3L1 cells at 1 µM with triplicate measurements, with 27 drugs validated as cytotoxic to one or both cell lines (Fig. 1; also see Additional File 1: Table S1).

**Figure 2:**
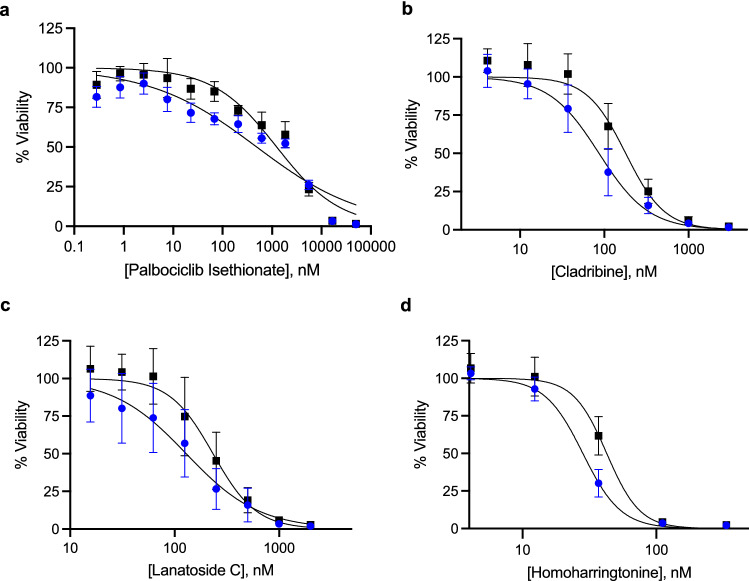
Drugs more cytotoxic towards CREB3L1-deficient HCC1806 cells (blue) as compared to CREB3L1 re-expressing HCC1806 + HACREB3L1 cells (black). Cells were plated and after 24 h were treated with the indicated concentration of drug, or solvent control, for 4 days. Solvents (max 0.4%) had little or no effect on the cell growth/number. Cells were stained, imaged and counted. Cell viability (%) was calculated as (# live cells in experimental well)/(# live cells in solvent control well)*100. Mean % viability ± SEM from triplicate measurements from at least 3 independent experiments. (**a**) Palbociclib Isethionate, 1:3 serial dilutions for concentrations 0–50 μM. (**b**) Cladribine, 1:3 serial dilutions for concentrations 0–9 μM. (**c**) Lanatoside C, 1:2 serial dilutions for concentrations 0–2 μM. (**d**) Homoharringtonine, 1:3 serial dilutions for concentrations 0–333 nM.

Drug Titration experiments were carried out on these 27 drugs to determine their EC_50_ values (i.e. the concentration of a drug necessary to reach half of the maximum response) in order to compare the efficacy of each drug (Fig. 1). The majority of drugs (20 drugs) were similarly cytotoxic to both cell lines (see Additional File 2: Fig. S1) or showed more cytotoxicity towards the HCC1806 + HACREB3L1 cells (3 drugs) (see Additional File 3: Fig. S2). There were 4 drugs that showed a preference for killing the more metastatic CREB3L1-deficient HCC1806 TNBC cells, palbociclib isethionate, cladribine, homoharringtonine and lanatoside C (Fig. 2, Table 1).

**Table 1:** EC_50_ values for CREB3L1-deficient HCC1806 cells, as compared to HCC1806 + HA-CREB3L1 cells.

Drug name	Drug target or class of agent	HCC1806 (EC_50_, nM)^a^	HCC1806 + HA-CREB3L1 (EC_50_, nM)^a^	EC_50_ range	p-value^b^	Comments
Homoharringtonine	Inhibits protein translation	29 ± 4	42 ± 7	Med nM	3.919E−04	**CREB3L1-Dependent**
Cladribine	Nucleoside Analog	92 ± 44	182 ± 60	Med nM	0.002	**CREB3L1-Dependent**
Lanatoside C	Na+/K+ ATPase	143 ± 85	233 ± 100	Med nM	0.056	**CREB3L1-Dependent**
Palbociclib Isethionate	CDK4/6	500 ± 130	1300 ± 490	High nM	1.882E−04	**CREB3L1-Dependent**
Sanguinarine Cl	STAT3, MMPs	540 ± 120	800 ± 200	High nM	0.249	CREB3L1-Independent
Zinc Pyrithione	Anti-bacterial, Anti-fungal	157 ± 62	266 ± 52	Med nM	0.319	Nearly CREB3L1-dependent
Hydroxy Camptothecin	Topoisomerase I	1.5 ± 0.2	1.3 ± 0.2	Low nM	0.130	CREB3L1-Independent
Irinotecan	Topoisomerase I	211 ± 43	116 ± 24	Med nM	0.028	CREB3L1 more sensitive
Teniposide	Topoisomerase II	74 ± 30	73 ± 13	Med nM	0.968	CREB3L1-Independent
Cephalomannine	Microtubules	3.8 ± 0.7	4.7 ± 1.6	Low nM	0.119	CREB3L1-Independent
Nocodazole	Tubulin and microtubules	27 ± 6	12 ± 1	Low nM	0.011	CREB3L1 more sensitive
Doxorubicin	Anthracycline	13 ± 4	17 ± 6	Low nM	0.244	CREB3L1-Independent
Doxorubicin HCl	Anthracycline	36 ± 11	51 ± 5	Med nM	0.102	CREB3L1-Independent
Daunorubicin HCl	Anthracycline	34 ± 6	32 ± 2	Med nM	0.591	CREB3L1-Independent
Cyclocytidine HCl	Nucleotide Analog	32 ± 8	40 ± 5	Med nM	0.241	CREB3L1-Independent
Carfilzomib	Proteasome	8.3 ± 0.5	7.7 ± 0.5	Low nM	0.235	CREB3L1-Independent
Bortezomib	20S proteasome	12 ± 6	7.9 ± 3.6	Low nM	0.125	CREB3L1-Independent
MLN9708	20S proteasome	85 ± 12	91 ± 16	Med nM	0.530	CREB3L1-Independent
MLN2238	20S proteasome	251 ± 22	233 ± 9	Med nM	0.277	CREB3L1-Independent
Digitoxin	Na+/K+ ATPase	49 ± 11	48.8 ± 0.7	Med nM	0.989	CREB3L1-Independent
Ouabain Octahydrate	Na+/K+ ATPase	62 ± 8	54 ± 10	Med nM	0.344	CREB3L1-Independent
Digoxin	Na +/K+ ATPase	95 ± 31	113 ± 35	Med nM	0.531	CREB3L1-Independent
Panobinostat	HDACs	15 ± 5	12 ± 3	Low nM	0.009	CREB3L1 more sensitive
Romidepsin	HDACs	0.87 ± 0.07	0.92 ± 0.06	Low nM	0.370	CREB3L1-Independent
Belinostat	HDACs	131 ± 15	207 ± 85	Med nM	0.457	CREB3L1-Independent
Octenidine	Anti-infective	530 ± 70	630 ± 220	High nM	0.487	CREB3L1-Independent
Cobimetinib	MEK1 pathway	3400 ± 500	3100 ± 300	High nM	0.184	CREB3L1-Independent

Drugs with CREB3L1-dependent effects are in bold.

^a^Mean ± SD from at least 3 replicate measurements.

^b^p-value; student t-test for significance, HCC1806 + HA-CREB3L1 as compared to HCC1806.

The 27 drugs that were cytotoxic towards the TNBC HCC1806 (± HA-CREB3L1) cells can be divided into groups. Several of the drugs are known chemotherapy agents that are generally cytotoxic to most cells, including: topoisomerase inhibitors (hydroxy camptothecin, irinotecan, teniposide), nucleoside or nucleotide analogs (cladribine, cyclocytidine HCl), microtubule disruptors (cephalomannine, nocodazole) or anthracyclines (daunorubicin HCl, doxorubicin, doxorubicin HCl), known to block critical cell functions such as DNA replication. Several of the cytotoxic drugs were HDAC inhibitors (belinostat, panobinostat, romidepsin) capable of altering epigenetic regulation of gene expression. Others could regulate protein expression through inhibition of protein translation/synthesis (homoharringtonine) or proteasomal-mediated protein degradation (bortezomib, carfilzomib, MLN2238, MLN9708). There were several cardiac glycosidases (digitoxin, digoxin, lanatoside C, ouabain octahydrate), best known as Na+/K+ ATPase inhibitors. In addition, there were a few more selective inhibitors of the MEK1 pathway (cobimetinib), STAT3/MMPs (sanguinarine Cl), CDK4/6 (palbociclib isethionate) and two less characterized drugs with anti-infective (octenidine) and anti-bacterial/anti-fungal (zinc pyrithione) activities, the latter of which has recently been shown to have anti-cancer properties through a newly described proteasomal deubiquitinase inhibitor^19^.

### Evaluation of promising drugs in multiple breast cell lines

To assess the cytotoxicity towards a non-tumorigenic breast cell line MCF10A, we carried out similar cytotoxicity determinations as done with the HCC1806 TNBC cells (see Additional file 4: Fig. S3). MCF10A cells were very sensitive to palbociclib isethionate and lanatoside C, with much lower EC_50_ values than the TNBC HCC1806 cells (see Additional file 4: Fig. S3a, c). Since palbociclib is currently used clinically, this observed cytotoxicity may not preclude its use. Little or no cytotoxicity was observed when MCF10A cells were treated with cladribine (see Additional file 4: Fig. S3b), suggesting that it may not have adverse effects towards normal tissue. The cytotoxicity profile of homoharringtonine was similar for both non-tumorigenic MCF10A breast cells and TNBC HCC1806 cells (see Additional file 4: Fig. S3d).

**Figure 3:**
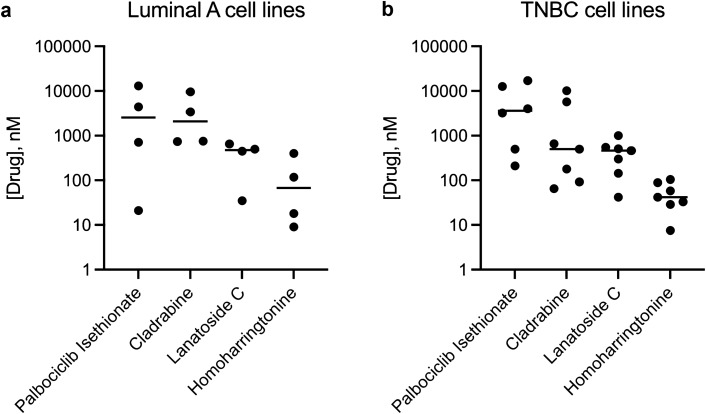
Drug sensitivity across a panel of CREB3L1-deficient luminal A and TNBC cell lines. Cell viability was measured after 4 days of drug treatment over a range of drug concentrations as before. EC_50_ values were determined as per Table 2 and plotted here for comparison.

We were particularly interested in the 4 promising drugs which showed more cytotoxic effects towards the more metastatic CREB3L1-deficient HCC1806 TNBC cells since these are typically the most challenging types of cancer cells to treat. To assess the utility of these drugs across additional CREB3L1-deficient breast cancer cells, we evaluated the cytotoxicity of palbociclib isethionate, cladribine, lanatoside C and homoharringtonine across a panel of CREB3L1-deficient TNBC cell lines, including BT549, HCC1395, HCC38, Hs578T, MDA-MB-157 and MDA-MB-436 (Table 2). Since luminal A breast cancers that are CREB3L1-deficient also show a poor prognosis as compared to those expressing CREB3L1^12^, we extended this analysis to determine the drug sensitivity in several luminal A breast cancer cell lines, some of which were also tested with re-expressed CREB3L1 (Table 2). HCC1428 luminal A breast cancer cells showed increased drug sensitivity to both palbociclib isethionate and cladribine as compared to the corresponding CREB3L1-expressing cells, however the opposite was true for lanatoside C and homoharringtonine. The TNBC HCC1806 cells exhibited a CREB3L1-dependent drug sensitivity for all four drugs. Thus, drug sensitivity may be impacted differently by CREB3L1 expression in different breast cancer cell lines, or in different breast cancer subtypes. Overall, a range of drug sensitivities were observed, but both luminal A and TNBC cells were most sensitive to homoharringtonine (Fig. 3).

**Table 2:** EC50 values across multiple CREB3L1-deficient TNBC and luminal A breast cancer cell lines.

Cell line	Palbociclib Isethionate		Cladribine		Lanatoside C		Homoharringtonine	
	Parental EC50, nM (n^a^)	Parental + HA-CREB3L1 EC50, nM (n^a^)	Parental EC50, nM (n^a^)	Parental + HA-CREB3L1 EC50, nM (n^a^)	Parental EC50, nM (n^a^)	Parental + HA-CREB3L1 EC50, nM (n^a^)	Parental EC50, nM (n^a^)	Parental + HA-CREB3L1 EC50, nM (n^a^)
BT549	12600 ± 4800 (9)		178 ± 84 (9)		42 ± 12 (9)		89 ± 10 (9)	
HCC1395	4000 ± 2000 (6)		10100 ± 1200 (6)		510 ± 300 (6)		104 ± 31 (6)	
HCC1806	500 ± 130 (9)	1300 ± 490 (9)	92 ± 44 (9)	182 ± 60 (9)	143 ± 85 (9)	233 ± 100 (9)	29 ± 4 (9)	42 ± 7 (9)
HCC38	210 ± 70 (9)		65 ± 56 (9)		300 ± 100 (9)		58 ± 10 (9)	
Hs578T	3200 ± 1400 (4)		5700 ± 1200 (4)		1000 ± 800 (4)		42 ± 24 (4)	
MDA-MB-157	ND		5000 ± 4300 (4)		550 ± 240 (4)		7.5 ± 1.2 (4)	
MDA-MB-436	17000 ± 14000 (4)		660 ± 180 (4)		460 ± 250 (4)		33 ± 36 (4)	
HCC1428	21 ± 15 (4)	160 ± 250 (4)	740 ± 330 (4)	2200 ± 1700 (4)	35 ± 8 (4)	32 ± 8 (4)	9 ± 3 (4)	10 ± 3 (4)
MCF7	4400 ± 3500 (4)		3400 ± 2100 (4)		450 ± 490 (4)		18 ± 9 (4)	
T47D	13000 ± 9800 (4)	7200 ± 9200 (4)	9600 ± 1800 (6)	1800 ± 1300 (6)	500 ± 50 (4)	89 ± 34 (4)	400 ± 180 (6)	88 ± 55 (6)
ZR-75-1	710 ± 350 (8)		750 ± 350 (9)		650 ± 510 (9)		117 ± 35 (9)	

n, number of replicate measurements.

^a^Cell viability was determined over a range of drug concentrations after 4 days of drug treatment and EC_50_ values were determined.

### Combining promising drugs with either paclitaxel or doxorubicin

Since new drug treatments are most likely to be offered in combination with existing therapies, we set out to evaluate possible synergistic effects between some of the currently used chemotherapy agents and the four most promising drugs identified. Cytotoxic chemotherapy agents frequently used for TNBC patients include the anthracycline doxorubicin and the microtubule stabilizing drug paclitaxel.

Doxorubicin and paclitaxel and were each tested separately, in pairwise combination drug titration experiments with each of palbociclib isethionate, cladribine, homoharringtonine and lanatoside C. Drug titration experiments were initially carried out for paclitaxel and doxorubicin individually in HCC1806 ± HA-CREB3L1 cells, to determine EC_50_ values for each drug when used alone (see Additional file 2 and 5: Figs S1, S4). EC_50_ values for doxorubicin were very similar for the two cell lines with 13 ± 4 nM for HCC1806 and 17 ± 6 nM for HCC1806 + HA-CREB3L1 (Table 1). EC_50_ values for paclitaxel were also very similar for the two cell lines with 1.2 ± 0.5 nM for HCC1806 and 1.2 ± 0.4 nM for HCC1806 + HA-CREB3L1 (see Additional file 5: Fig. S4).

**Figure 4:**
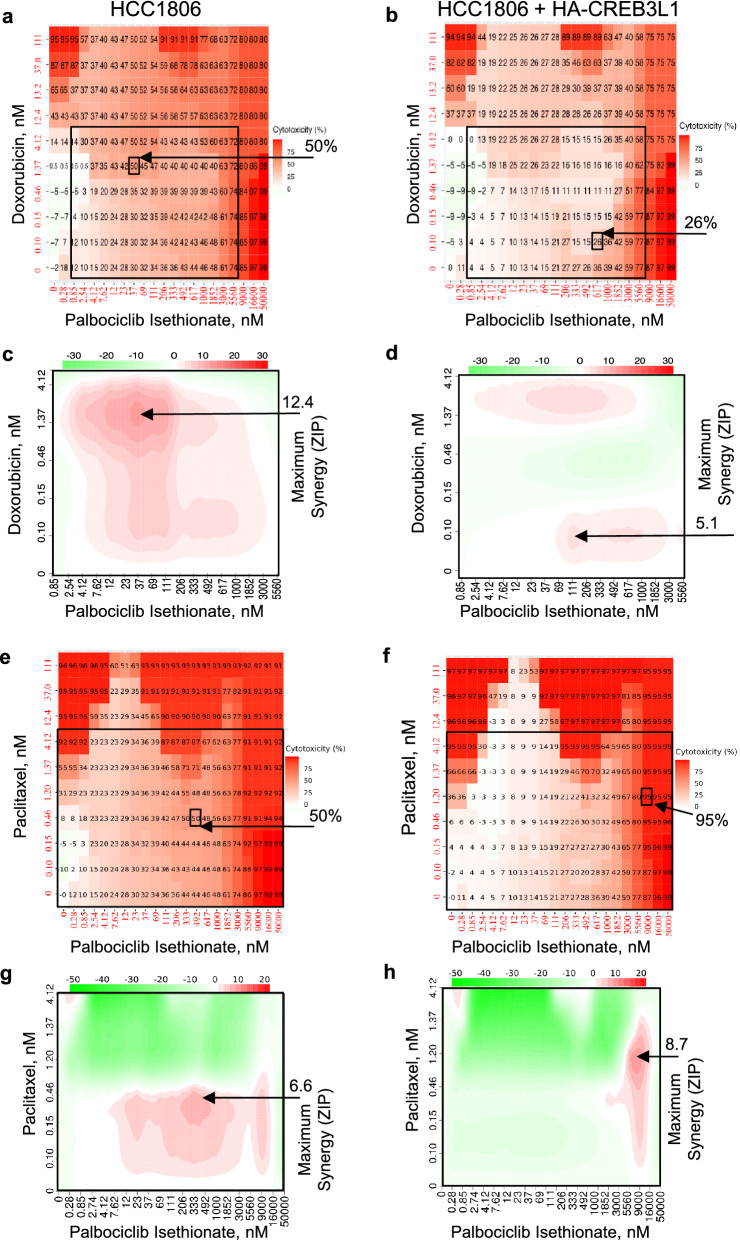
Assessing possible drug synergy between palbociclib isethionate and either doxorubicin (**a-d**) or paclitaxel (**e–h**). Cell viability (**a, b, e, f**) was measured after 4 days of drug treatment over a range of drug concentrations for HCC1806 (**a, c, e, g**) and HCC1806 + HA-CREB3L1 (**b, d, f, h**) cells. Palbociclib Isethionate EC_50_ for HCC1806 = 500 nM; 1:3 serial dilutions (0–9 mM). Doxorubicin EC_50_ for HCC1806 = 13 nM; 1:3 serial dilutions (0–111 nM). Paclitaxel EC_50_ for HCC1806 = 1.2 nM; 1:3 serial dilutions (0–111 nM). A zero-interaction potency score (ZIP; **c, d, g, h**) was calculated by the SynergyFinder R-package for each data point to measure possible drug synergy; ZIP > 10 indicates synergy.

**Figure 5:**
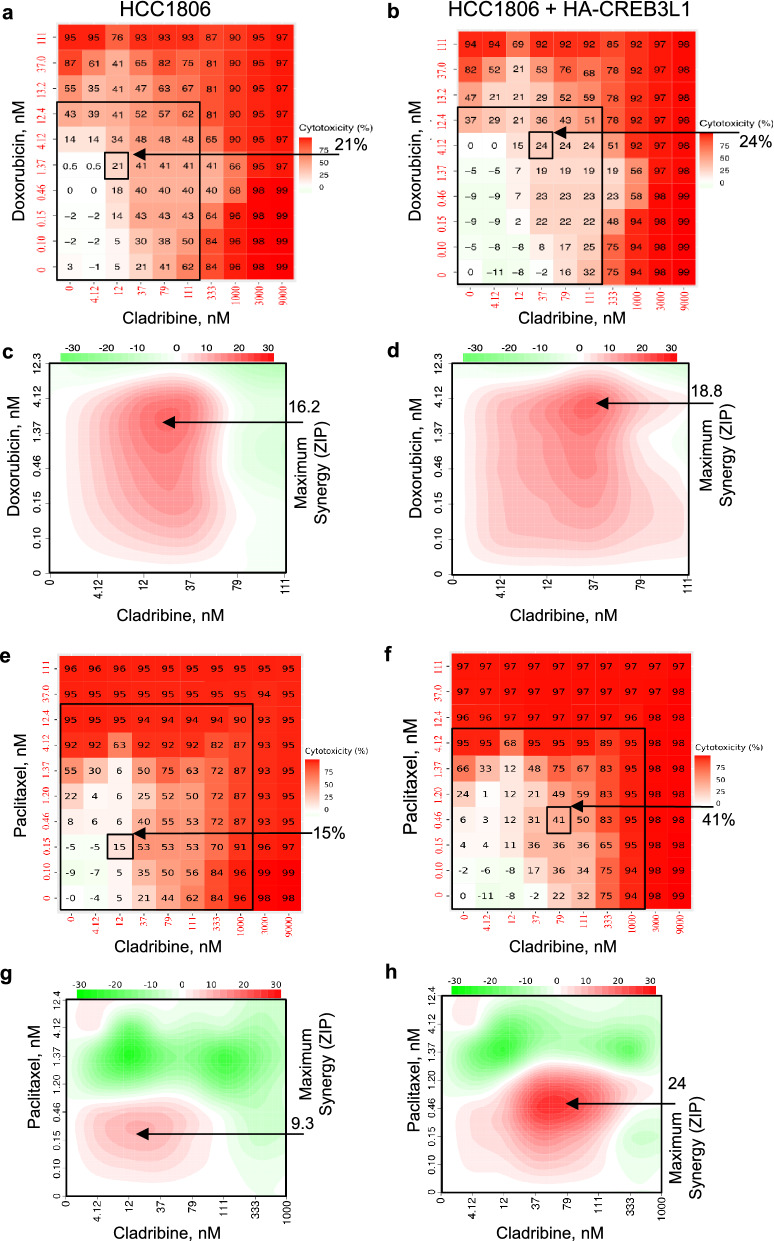
Assessing possible drug synergy between cladribine and either doxorubicin (**a-d**) or paclitaxel (**e–h**). Cell viability (**a, b, e, f**) was measured after 4 days of drug treatment over a range of drug concentrations for HCC1806 (**a, c, e, g**) and HCC1806 + HA-CREB3L1 (**b, d, f, h**) cells. Cladribine EC_50_ for HCC1806 = 92 nM; 1:3 serial dilutions (0–9 mM). Doxorubicin EC_50_ for HCC1806 = 13 nM; 1:3 serial dilutions (0–111 nM). Paclitaxel EC_50_ for HCC1806 = 1.2 nM; 1:3 serial dilutions (0–111 nM). A zero-interaction potency score (ZIP; **c, d, g, h**) was calculated by the SynergyFinder R-package for each data point to measure possible drug synergy; ZIP > 10 indicates synergy.

**Figure 6:**
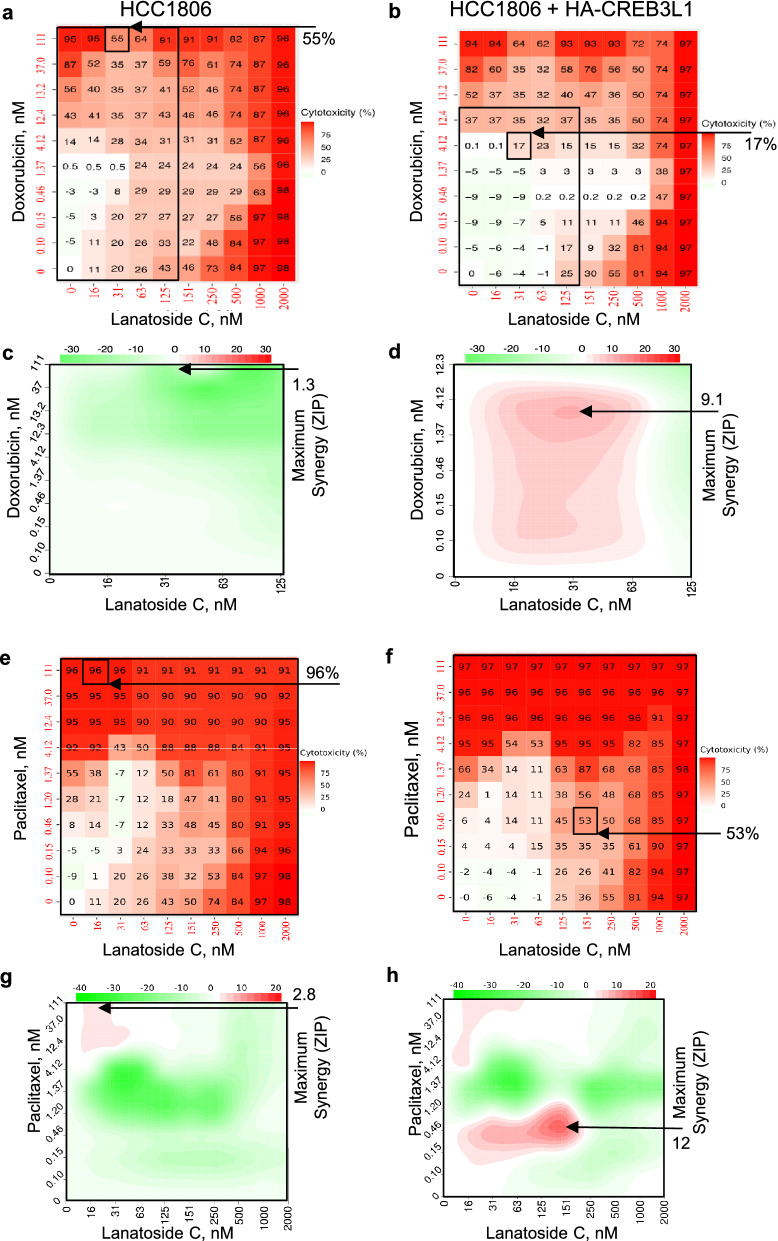
Assessing possible drug synergy between lanatoside C and either doxorubicin (**a-d**) or paclitaxel (**e–h**). Cell viability (**a, b, e, f**) was measured after 4 days of drug treatment over a range of drug concentrations for HCC1806 (**a, c, e, g**) and HCC1806 + HA-CREB3L1 (**b, d, f, h**) cells. Lanatoside C EC_50_ for HCC1806 = 143 nM; 1:2 serial dilutions (0–2 mM). Doxorubicin EC_50_ for HCC1806 = 13 nM; 1:3 serial dilutions (0–111 nM). Paclitaxel EC_50_ for HCC1806 = 1.2 nM; 1:3 serial dilutions (0–111 nM). A zero-interaction potency score (ZIP; **c, d, g, h**) was calculated by the SynergyFinder R-package for each data point to measure possible drug synergy; ZIP > 10 indicates synergy.

**Figure 7:**
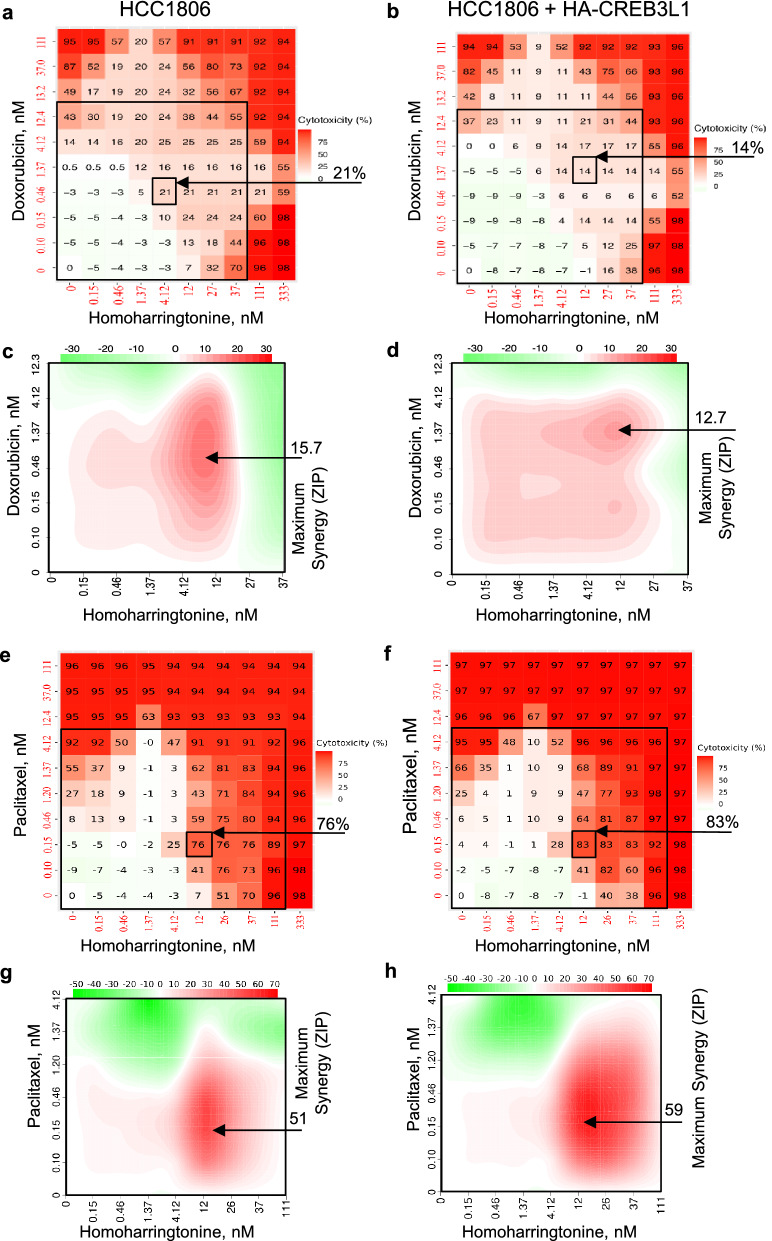
Assessing possible drug synergy between homoharringtonine and either doxorubicin (**a-d**) or paclitaxel (**e–h**). Cell viability (**a, b, e, f**) was measured after 4 days of drug treatment over a range of drug concentrations for HCC1806 (**a, c, e, g**) and HCC1806 + HA-CREB3L1 (**b, d, f, h**) cells. Homoharringtonine EC_50_ for HCC1806 = 29 nM; 1:3 serial dilutions (0–333 nM). Doxorubicin EC_50_ for HCC1806 = 13 nM; 1:3 serial dilutions (0–111 nM). Paclitaxel EC_50_ for HCC1806 = 1.2 nM; 1:3 serial dilutions (0–111 nM). A zero-interaction potency score (ZIP; **c, d, g, h**) was calculated by the SynergyFinder R-package for each data point to measure possible drug synergy; ZIP > 10 indicates synergy.

We used a cross-design combination experiment as detailed in the methods where drug 1 is tested over a range of concentrations and drug 2 is maintained at its EC_50_ value. A second set of experiments then tested drug 2 over a range of concentrations and drug 1 is maintained at its EC_50_ value. Cells were treated for four days and cell viability assessed as before. The SynergyFinder r package was used to determine the possible additive, synergistic, or antagonistic effect of the combined drugs^18^. This recently developed synergy model improves upon previous scoring models including Highest simple agent, Lowe additivity and Bliss models^20^. The SynergyFinder package characterizes a synergy landscape of drug interaction by calculating a Zero Interaction Potency (ZIP) score defining delta scores for every input data point and interpolating untested data points in between^18^. The ZIP defined delta score is the additional response (% Inhibition) observed beyond the expected effect (as determined by the ZIP model) for the given concentrations of two drugs^20^. For example, a positive ZIP defined delta score of 10 (red in the synergy landscape) indicates that the response observed had a 10% higher inhibition than would be expected if the combined drug effect was non-interactive or additive, with values > 10 indicating significant synergy^20^ (Figs. 4, 5, 6 and 7; Table 3).

**Table 3:** Synergy of drug combinations.

Drug 1 (EC_50_^a^)	Drug 2 (EC_50_^a^)	HCC1806		HCC1806 + HA-CREB3L1	
		ZIP^b^ (drug1; drug2)	% Cytotoxicity	ZIP^b^ (drug1; drug2)	% Cytotoxicity
Palbociclib Isethionate (500 nM)	Doxorubicin (13.2 nM)	**12.4** (37 nM; 1.37 nM)	50	5.1 (111 nM; 0.08 nM)	26
Palbociclib Isethionate (500 nM)	Paclitaxel (1.2 nM)	6.6 (492 nM; 0.46 nM)	50	8.7 (9000 mM; 1.2 nM)	95
Cladribine (92 nM)	Doxorubicin (13.2 nM)	**16.2** (12 nM; 1.37 nM)	21	**18.8** (37 nM; 4.12 nM)	24
Cladribine (92 nM)	Paclitaxel (1.2 nM)	9.3 (12 nM; 0.15 nM)	15	**24** (79 nM; 0.46 nM)	41
Lanatoside C (143 nM)	Doxorubicin (13.2 nM)	1.3 (31 nM; 111 nM)	55	9.1 (31 nM; 4.12 nM)	17
Lanatoside C (143 nM)	Paclitaxel (1.2 nM)	2.8 (16 nM; 111 nM)	96	**12** (151 nM; 0.46 nM)	53
Homoharringtonine (29 nM)	Doxorubicin (13.2 nM)	**15.7** (12 nM; 0.46 nM)	21	**12.7** (12 nM; 1.37 nM)	14
Homoharringtonine (29 nM)	Paclitaxel (1.2 nM)	**51** (12 nM; 0.15 nM)	76	**59** (12 nM; 0.15 nM)	83

Significant values are in [bold].

^a^EC_50_ as single agent in HCC1806 cells.

^b^ZIP, zero-interaction potency; scores > 10 indicate synergy.

Some synergy was observed using palbociclib isethionate and doxorubicin for the CREB3L1-deficient HCC1806 cells, with a maximum ZIP synergy score of 12.4 giving rise to 50% cytotoxicity (Fig. 4a, c; Table 3). Synergy was not observed for these two drugs for the HCC1806 + HA-CREB3L1 cells, suggesting that the synergy is CREB3L1-deficiency dependent (Fig. 4b, d; Table 3). A strong antagonistic effect was observed at 111 nM paclitaxel and 12, 23, and 37 nM palbociclib, suggesting they are less cytotoxic when used at these concentrations (Fig. 4e, f). No positive ZIP synergy scores above 10 were observed for the palbociclib and paclitaxel drug combination (Fig. 4e–h; Table 3).

Cladribine exhibited synergistic cytotoxicity when combined with doxorubicin in both cell lines, suggesting CREB3L1-independent synergy, with ZIP scores of 16.2 and 18.8, with corresponding cytotoxicity of 21% and 24% for HCC1806 and HCC1806 + HA-CREB3L1 cells, respectively (Fig. 5a–d; Table 3). Cladribine was also selectively synergistic with paclitaxel but only for HA-CREB3L1 expressing cells and not the HCC1806 cells (Fig. 5e–h; Table 3). In contrast, lanatoside C did not display any synergy with doxorubicin but was synergistic towards HCC1806 + HA-CREB3L1 cells when combined with paclitaxel (Fig. 6, Table 3).

The strongest synergy was exhibited by homoharringtonine, which showed similar effects in HCC1806 ± HA-CREB3L1 cells, suggesting CREB3L1-independent effects (Fig. 7, Table 3). When paired with doxorubicin, the ZIP synergy scores for homoharringtonine were 15.7 and 12.7, however this resulted in fairly low cell cytotoxicity of 21% and 14%. When homoharringtonine and paclitaxel were used in combination, they provided a very high ZIP synergy score of 51 and 59, and a corresponding high cytotoxicity of 76% and 83% (Fig. 7; Table 3). The most synergistic drug combination was achieved using 12 nM homoharringtonine and 0.15 nM paclitaxel, resulting in 76–83% cytotoxicity (Table 3). These drug doses were considerably lower than the EC_50_ values of individual drugs when used alone, with homoharringtonine (EC_50_ = 29 nM) and paclitaxel (EC_50_ = 1.2 nM). These promising results suggest that homoharringtonine and paclitaxel combination treatment could be very effective in treating TNBCs using relatively low concentrations such that toxic side-effects would be minimized.

## Discussion

CREB3L1 is a metastasis suppressor in breast cancer and low CREB3L1 expression is associated with poor prognosis in both TNBC and luminal A breast cancers^12^. Since loss of CREB3L1 is prevalent in ~ 75% of TNBCs and this loss contributes to metastatic breast cancer cell properties^11,15^, we focused on identifying compounds most cytotoxic towards the more metastatic CREB3L1-deficient TNBCs. However, the drug screen also identified various compounds that were very cytotoxic towards both HCC1806 ± HA-CREB3L1, independent of CREB3L1 expression. Many of these were known cytotoxic chemotherapy agents including inhibitors of: topoisomerases (hydroxy camptothecin, irinotecan, teniposide), microtubules (cephalomannine, nocodazole), proteasomes (carfilzomib, bortezomib, MLN9708, MLN2238), histone deacetylation (HDAC; panobinostat, romidepsin, belinostat), and DNA replication (doxorubicin, doxorubicin HCl, daunorubicin HCl, cyclocytidine HCl). Other cytotoxic drugs identified had defined molecular targets such as: STAT3/MMPs (sanguinarine Cl), MEK1 (cobimetinib), or Na+/K+ ATPase (digitoxin, ouabain octahydrate, digoxin). Yet others had poorly defined targets in mammalian cells, being better known for their anti-bacterial/anti-fungal (zinc pyrithione) or anti-infection (octenidine) functions. These latter two groups may be of particular interest for future studies since less is known about their potential use as anti-cancer agents.

We chose to focus on the 4 compounds that were more cytotoxic towards HCC1806 cells as compared to HCC1806 + HA-CREB3L1: palbociclib isethionate, lanatoside C, cladribine and homoharringtonine. Palbociclib isethionate is a highly specific cyclin-dependent kinase 4 (CDK4) and CDK6 inhibitor and is currently under investigation in a phase I/II nonrandomized, open-label, single-arm trial in combination with bicalutamide (a non-steroidal androgen receptor inhibitor) for safety and efficacy in TNBC^21,22^. Our results showed that both luminal A and TNBC cells required low micromolar concentrations to achieve cytotoxicity across a panel of cell lines, and it displayed modest synergy when combined with doxorubicin. Currently, combining CDK4/6 inhibitors with hormonal treatments is indicated for hormone receptor positive, HER2 negative breast cancer. CDK4/6 inhibitors show promise as potential biomarkers because these targets are frequently amplified in breast cancer and specifically inhibiting these targets might produce selective antiproliferative activity in TNBC cells^21,23^. Palbociclib is highly selective and is thought to rely on the Rb1 pathway to provide a G1 block, inhibiting cells from entering S phase, thereby preventing cell growth and DNA replication^24^. This inhibitor prevents the phosphorylation of Rb by CDK4 and CDK6, which normally promotes DNA replication and therefore cell division^25^. Many CDK inhibitors actually favor CDK1 and CDK2 inhibition even though CDK4 and CDK6 have been identified as the most important CDKs for regulating cell proliferation^21,24^. Palbociclib is especially effective in Rb+ breast cancers, including advanced hormone receptor positive, Rb+ breast cancers^26^.

Interestingly, the activin-SMAD pathway, a downstream target of CDK4/6 (independent of Rb) was shown to be a good target for palbociclib in CREB3L1-deficient T47D luminal A breast cancer cells^27^. Therefore, it is possible that in CREB3L1-deficient TNBC there are mediators of cytostasis (independent of Rb), downstream of CDK4/6, like the activin-SMAD pathway. Inhibition of CDK4/6 by palbociclib worked together with SMAD signaling in T47D cells to prevent cell division^27^. The SMAD pathway promotes cytostasis, enhanced by palbociclib in ER + T47D cells, but in aggressive CREB3L1-deficient (Hs578T) cells this pathway possibly promotes tumorigenesis, and the way in which the activin-SMAD pathway interacts with CDKs in this context is unknown^27^. The modest synergy we observed with the combination palbociclib isethionate and doxorubicin suggests this combination could be worth pursuing.

Lanatoside C is a cardiac glycoside and inhibits the alpha subunit of the Na^+^/K^+^-ATPase and has recently been shown to induce apoptosis selectively in breast, lung and liver cancer cells^28^. Cardiac glycosides are indicated in the treatment of cardiovascular disease and increase cardiac output by indirectly increasing intracellular calcium of cardiomyocytes through the inhibition of the Na+/K+ -ATPase. In lung, liver and breast cancer cells (MCF7; luminal A and CREB3L1-deficient), lanatoside C was shown to selectively kill cancer cells by arresting the cells in G2 and M phase, likely through effects on the JAK/STAT and PTEN/p53 signaling pathways^28^. In hepatocarcinoma, lanatoside C was found to act through PKCδ to induce apoptosis^29^. The possible role of CREB3L1 in regulating these pathways is not currently known.

Cladribine is a cytotoxic purine analog and it has previously been demonstrated to be highly cell specific, producing less toxic side effects as a result^30^. Cladribine specifically targets lymphocytes and produces a remarkably strong clinical response in hairy-cell leukemia, chronic lymphocytic leukemia, and non-Hodgkin’s lymphoma^30,31^. Lymphocytes are unique in that they express high levels of DCK^30,31^. Cladribine is a prodrug and requires phosphorylation by DCK to generate its active form, 2-chlorodeoxyadenosine triphosphate^31^, and this requirement fulfilled by the high levels of DCK in lymphocytes is the likely attribute of its high cellular specificity. Once active, cladribine competes with dATP for incorporation into DNA and also potently inhibits ribonucleotide reductase, interfering with nucleotide metabolism^31,32^.

DCK is also overexpressed in poor outcome breast cancers, including the metastatic CREB3L1-deficient breast cancer cell lines (HCC1954, BT474, SK-BR-3, MDA-MB-231, MCF7, and T47D) and has low expression in the non-tumorigenic breast cell line MCF10A^12,33^. This may explain the specific cytotoxic effects of cladribine in the CREB3L1-deficient TNBC cell line HCC1806 and not in the same cell line expressing CREB3L1 or the MCF10A cells. Similarly, downregulation of DCK is likely the major contributor to cladribine resistance^34^ and downregulation of DCK in breast cancer cells also likely confers resistance to another nucleoside analog, gemcitabine^35^.

Apoptosis is regulated by the balance of cell survival and proapoptotic proteins. Cancer cells are under oncogenic and metabolic stress which requires the inhibition of intrinsic apoptotic pathways to allow for cell survival^36^. Anti-apoptotic proteins such as MCL-1, BCL-2 and BCL-xL prevent apoptosis by sequestering the pro-apoptotic family members. MCL-1 is frequently amplified and the protein overexpressed in breast cancers^37–39^. Inhibitors of MCL-1 and MCL-1 knockdown reduce the cell viability in vitro and restrict TNBC cell growth in vivo, suggesting TNBCs are particularly dependent upon MCL-1 for cell survival^36,39–41^.

Homoharringtonine blocks protein synthesis by transiently binding to ribosomes and inhibiting protein translation^42^. This results in a selective loss of proteins with short half-lives, such as those regulating cell proliferation and cell survival^43,44^. Homoharringtonine has been shown to rapidly reduce the expression of several anti-apoptotic proteins, including MCL-1, BCL-2 and survivin in several TNBC cell lines^44^. Thus, the cytotoxicity we observed is likely due to the loss of MCL-1 expression causing apoptosis.

Our results are consistent with a genome-wide siRNA lethality screen that identified a large overrepresentation of proteasome genes as a specific dependency in basal-like TNBCs and this was shown to be mediated by NOXA and MCL-1^45^. Proteasome inhibition was further shown to reduce the growth of basal-like TNBC tumors in mouse xenograft studies^45^. Similar results were seen upon treatment of MDA-MB-231 TNBC xenografts with homoharringtonine with reduced tumor growth without general toxicity for the mice^44^.

Overexpression of MCL-1 in several TNBC cell lines has been shown to increase resistance to the chemotherapy agent’s doxorubicin and docetaxel^40^. The MCL-1 inhibitor S63845 displayed synergistic activity with docetaxel in TNBC, suggesting that blocking MCL-1 function may sensitize cells to cytotoxic chemotherapeutic agents by reducing this pro-survival activity^40^. MCL-1 levels can be upregulated even higher in breast tumor samples from patients treated with a variety of neoadjuvant chemotherapy agents including doxorubicin, cyclophosphamide and fluorouracil^38^. These results suggest that increased MCL-1 expression protects TNBCs from chemotherapy-induced apoptosis^38^. Our results suggest that homoharringtonine may similarly reduce the levels of MCL-1 and sensitize TNBC cells to the cytotoxic agent paclitaxel. The strong synergy observed for the homoharringtonine—paclitaxel combination treatment suggests it may be both effective and have reduced toxic side-effects, an ideal combination for patients with TNBC.

In addition, paclitaxel binds reversibly to ß-tubulin, interfering with microtubule polymerization, and repressing the cell cycle at the G2-M stage^46^. Paclitaxel can also cause improper chromosome segregation during mitosis, resulting in arrest during cell growth and cell death^47^. Since homoharringtonine blocks protein translation, the levels of short-lived cell cycle proteins such as c-Myc, cyclin D1 and Cdk2 can also be reduced^48^. Thus, the combination of paclitaxel and homoharringtonine may slow or block progression through the cell cycle, giving rise to increased cell death, consistent with the observed increase in cytotoxicity observed when the two drugs are used together.

## Conclusions

Our large scale drug screen identified a number of compounds that are cytotoxic towards TNBC cells. The most effective of these was the protein translation inhibitor homoharringtonine, which proved to be highly synergistic when combined with paclitaxel. These results suggest that TNBC cells may be particularly sensitive to combination homoharringtonine—paclitaxel treatment and that effective TNBC cell killing may be achieved using considerably lower drug concentrations for each, reducing their toxic side-effects yet maintaining their anti-cancer efficacy.

## Supplementary Information

Supplementary Table.

Supplementary Figure 1.

Supplementary Figure 2.

Supplementary Figure 3.

Supplementary Figure 4.

## Data Availability

Data and materials available upon request to DHA.

## Abbreviations

CREB3: cAMP responsive element binding protein 3
CREB3L1: CREB3-like 1
DMF: Dimethylformamide
DMSO: Dimethyl sulfoxide
ER: Estrogen receptor
EtOH: Ethanol
HA: Hemagglutinin
HER2: Human epidermal growth factor receptor 2
PBS: Phosphate buffered saline
PR: Progesterone receptor
RFP: Red fluorescent protein
TNBC: Triple-negative breast cancer
ZIP: Zero Interaction Potency
