# Supplementary material for: Homoharringtonine demonstrates a cytotoxic effect against triple-negative breast cancer cell lines and acts synergistically with paclitaxel
Source: Sci Rep. 2022 Sep 19;12:15663. doi: 10.1038/s41598-022-19621-7 (PMC9485251; doi:10.1038/s41598-022-19621-7)
Supplement: Supplementary file 5 — Supplementary Figure 4. [file 41598_2022_19621_MOESM5_ESM.pdf]

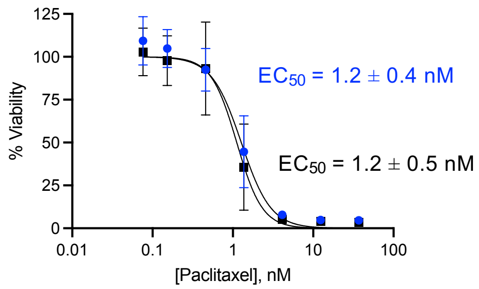

Additional File 5: Figure S4: Paclitaxel sensitivity for HCC1806 +HACREB3L1 cells (black) and CREB3L1-deficient HCC1806 cells (blue). Cells were plated and after 24 hours were treated with the indicated concentration of drug, or solvent control, for 4 days. Solvents (max 0.4%) had little or no effect on the cell growth/number. Cells were stained, imaged and counted. Cell viability (%) was calculated as (# live cells in experimental well) / (# live cells in solvent control well)*100. Mean % viability ± SEM from duplicate or triplicate measurements from at least 3 independent experiments. 1:3 serial dilutions for concentrations 0-111 nM.
